# Near-infrared spectroscopy as a tool for *in vivo* analysis of human muscles

**DOI:** 10.1038/s41598-019-44896-8

**Published:** 2019-06-13

**Authors:** Antonio Currà, Riccardo Gasbarrone, Alessandra Cardillo, Carlo Trompetto, Francesco Fattapposta, Francesco Pierelli, Paolo Missori, Giuseppe Bonifazi, Silvia Serranti

**Affiliations:** 1 Academic Neurology Unit, A. Fiorini Hospital, Terracina (LT), Department of Medical-Surgical Sciences and Biotechnologies, Sapienza University of Rome, Polo Pontino, Via Firenze snc, 04019 Terracina, LT Italy; 2 Research Center for Biophotonics, Sapienza University of Rome, Polo Pontino, Corso della Repubblica 79, 04100 Latina, Italy; 3 Department of Chemical Engineering, Materials & Environment, Sapienza University of Rome, Via Eudossiana, 18 - 00184 Roma, Italy; 4 IRCCS Ospedale Policlinico San Martino, and Department of Neuroscience, Rehabilitation, Ophthalmology, Genetics, Maternal and Child Health, University of Genova, Largo Rosanna Benzi 10, 16132 Genova, Italy; 5 Neurology Unit, Policlinico Umberto I, Department of Human Neurosciences, Sapienza University of Rome, Via dell’Università 30, 00185 Roma, Italy; 6 IRCCS Neuromed, and Academic Neuro-Rehabilitation Unit, ICOT, Latina, Department of Medical-Surgical Sciences and Biotechnologies, Sapienza University of Rome, Polo Pontino, Via Franco Faggiana 1668, 04100 Latina, Italy; 7 Neurosurgery Unit, Policlinico Umberto I, Department of Human Neurosciences, Sapienza University of Rome, Viale del Policlinico 155, 00161 Roma, Italy

**Keywords:** Translational research, Optical materials and structures

## Abstract

Recent advances in materials and fabrication techniques provided portable, performant, sensing optical spectrometers readily operated by user-friendly cabled or wireless systems. Such systems allow rapid, non-invasive, and not destructive quantitative analysis of human tissues. This proof-of-principle investigation tested whether infrared spectroscopy techniques, currently utilized in a variety of areas, could be applied in living humans to categorize muscles. Using an ASD FieldSpec® 4 Standard-Res Spectroradiometer with a spectral sampling capability of 1.4 nm at 350–1000 nm and 1.1 nm at 1001–2500 nm, we acquired reflectance spectra in visible short-wave infra-red regions (350–2500 nm) from the upper limb muscles (flexors and extensors) of 20 healthy subjects (age 25–89 years, 9 women). Spectra off-line analysis included preliminary preprocessing, Principal Component Analysis, and Partial Least-Squares Discriminant Analysis. Near-infrared (NIR) spectroscopy proved valuable for noninvasive assessment of tissue optical properties *in vivo*. In addition to the non-invasive detection of tissue oxygenation, NIR spectroscopy provided the spectral signatures (ie, “fingerprints”) of upper limb flexors and extensors, which represent specific, accurate, and reproducible measures of the overall biological status of these muscles. Thus, non-invasive NIR spectroscopy enables more thorough evaluation of the muscular system and optimal monitoring of the effectiveness of therapeutic or rehabilitative interventions.

## Introduction

Since the invention of the muscle oximeter by Glenn Millikan in the 1940s^1^, the application of optical methods has contributed extensively and significantly to the study of human tissues *in vivo*. In particular, near infrared (NIR) spectroscopy has been used for decades in health and medicine to measure tissue oxygenation and to detect hemoglobin and myoglobin content of skeletal muscle; there are clinical devices on the market that are used to monitor patients in hospitals today^2^. The success of NIR spectroscopy stems from various factors. First, human tissues are relatively transparent to light in the NIR spectral window; second, NIR light is either absorbed by pigmented compounds or scattered in tissues^3^; third, NIR light penetrates human tissues, because the dominant factor in its tissue transport is scattering^4^; and fourth, the high attenuation of NIR light in tissue is mainly due to a specific chromophore (i.e., hemoglobin). Because this oxygen-transporting protein contained in red blood cells is best detected in the capillary vessels of microcirculation (<1 mm diameter)^3^, using transillumination spectroscopy in tissues sufficiently transparent in the NIR range enables real-time non-invasive detection of hemoglobin oxygenation^5^. The ability to obtain blood oxygenation level-dependent data launched NIR spectroscopy in functional studies of the brain in the early 1990s^6^.

Apart from the medical and functional applications described above, optical spectroscopy and related spectra chemometrics are widely applied to perform both qualitative and quantitative analysis. Near Infrared Reflectance Spectroscopy (NIRS)-based investigation is non-invasive and not destructive, a feature ensuring that over the last 40 years, NIRS emerged progressively as a rapid method for testing the quality of intact samples from the light they reflect^7,8^. NIRS is now considered one of the best means for achieving quality control effectively and conveniently^9,10^. In the food industry, where determination of authenticity and detection of adulteration exerts relentless pressure, the combined application of NIRS-based detection and chemometrics allows prediction of chemical constituents in animal meats^11^, classification of fresh and frozen-thawed pork muscles^12,13^, and discrimination of lamb muscles^14^.

Dramatic advancements in technology and device miniaturization have further boosted NIRS applications; at present, they can be applied using devices that are cost-effective, small-sized, portable, simple and quick to use to build characteristic spectra that represent the “fingerprint” of examined samples^8^. This ability has opened the way of using spectra as surrogate markers of complex attributes of organic structures, which can be studied and classified with the application of specific statistical packages, without the need for chemical information from the analysis of molecular bonds in the NIR spectrum. At present, this application of NIR spectroscopy is used to perform systematic environmental remote and proximal sensing^15^, specifically in the primary/secondary raw materials sector^16–18^, cultural heritage^19,20^, the agricultural/food industry^21–23^, the pharmaceutical and chemical industry^24,25^, and analytical science^26^.

NIRS applications in food industry revealed that muscle tissue has optical properties suitable for spectral analysis and classification; we reasoned that living human muscles might also profit from undergoing optical spectroscopy^27^. At present, a cheap, reliable, and widely applicable technique for non-invasive *in vivo* analysis of human muscles is lacking, and we wished to determine whether NIRS of muscles can be adopted in clinical investigation without significant cost and time penalties.

We are currently unaware of any a priori knowledge about chemometrics applied to NIR spectra acquired *in vivo* from human muscles; therefore, this study essentially provided an exploratory approach. At this stage of the research, we were not interested in gaining information about the intimate physical chemical composition of living muscles. Rather, we sought to obtain an objective indicator of the current state of the organ that could be measured accurately and observed reproducibly from outside the organ itself. Therefore, in this study we investigated the reliability and accuracy of the visible and NIRS application to identify and authenticate muscle groups in the upper limb, without depending on chemical information. We acquired spectra to check whether they provide muscles’ “fingerprints”, whether these “fingerprints” can be modeled and classified, and whether they change according to anthropometric or physiologic variables.

To address these issues, we performed serial experiments in normal subjects aimed to collect the reflectance spectra acquired from the ventral and dorsal sides of the arm, a body segment that houses two different muscle groups: flexors (biceps and brachialis) and extensors (triceps). We used a portable visible short-wave infrared (Vis-SWIR) spectroradiometer – a device operating in the 350–2500 nm wavelength range – and applied chemometric techniques for exploring data and setting up a model to individuate muscles. Therefore, we used statistical methods for enucleating differences between the acquired spectra (chemometric analysis) and to define classification models that enabled Vis-SWIR spectroscopy to distinguish the group muscles of the arm.

## Results

### Exploratory analysis

**Figure 1:**
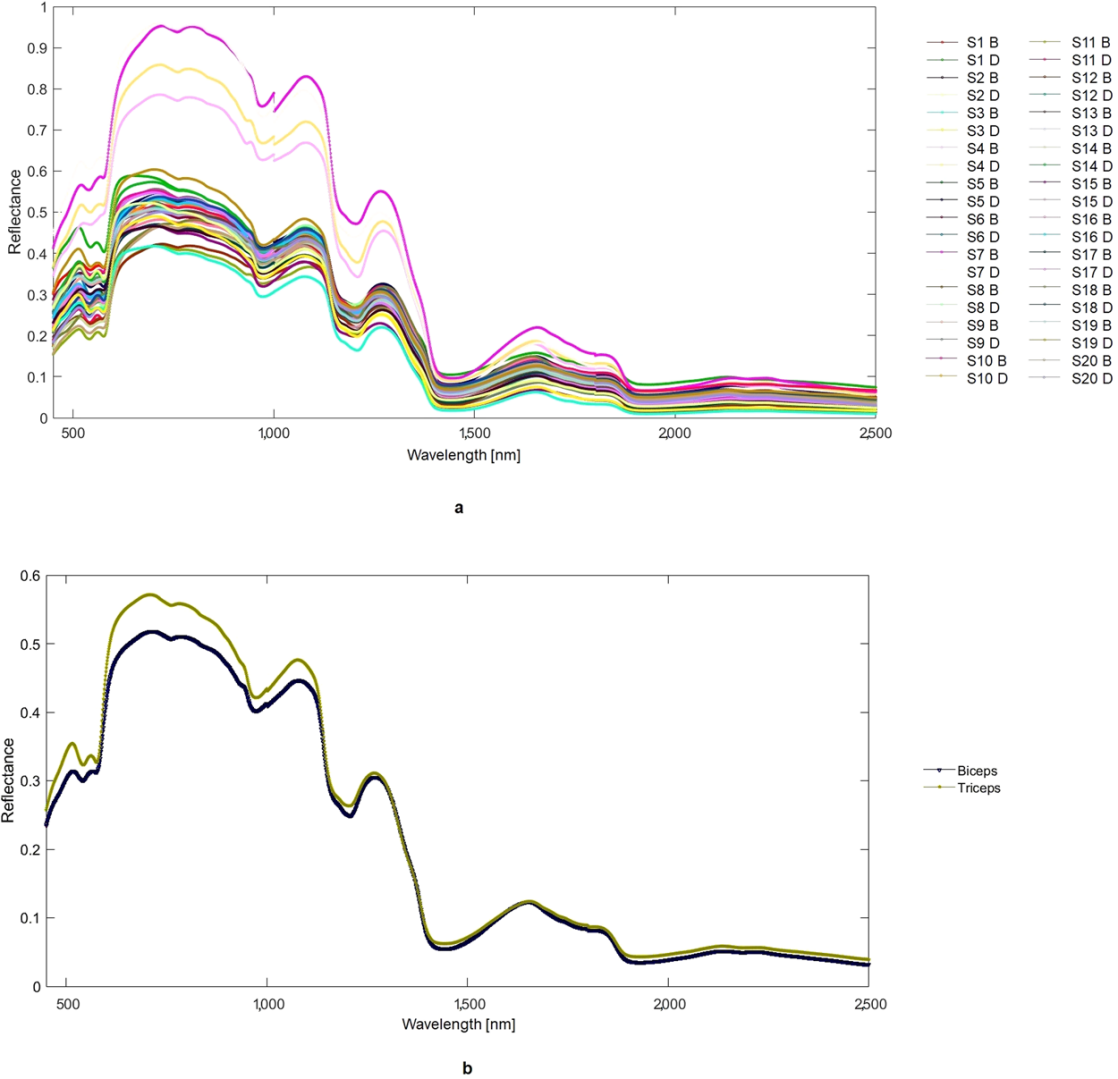
(**a**) Mean reflectance spectra of the ventral and dorsal arm for each subject. (**b**) Mean reflectance spectra of ventral arm/biceps (blue) and dorsal arm/triceps (yellow) averaged over all subjects. In the legend, the first letters identify the subject. D, dorsal arm; B, ventral arm.

Raw mean spectra of the “ventral arm” and “dorsal arm” were collected for each subject (n = 50/side/subject, total 2000), and grand averages were computed for all subjects (Fig. 1a,b).

Visual inspection of the grand averages revealed that spectra acquired from ventral and dorsal arms differ. The amplest divergence in spectra can be seen at wavelengths around 760 nm, 970 nm, 1200 nm, and 1440 nm. These wavelengths correspond to light-adsorbing groups H_2_O, CH, or CH_2_ (762 nm); H_2_O or CH (973); CH (1206 nm); H_2_O, CH, ROH, CONH_2_, or CONHR (1442 nm); CH (1796 nm); H_2_O, RCO_2_R, or CONH_2_ (1930 nm); and RNH_2_, CHC, or CC (2186 nm)^9,14^.

**Figure 2:**
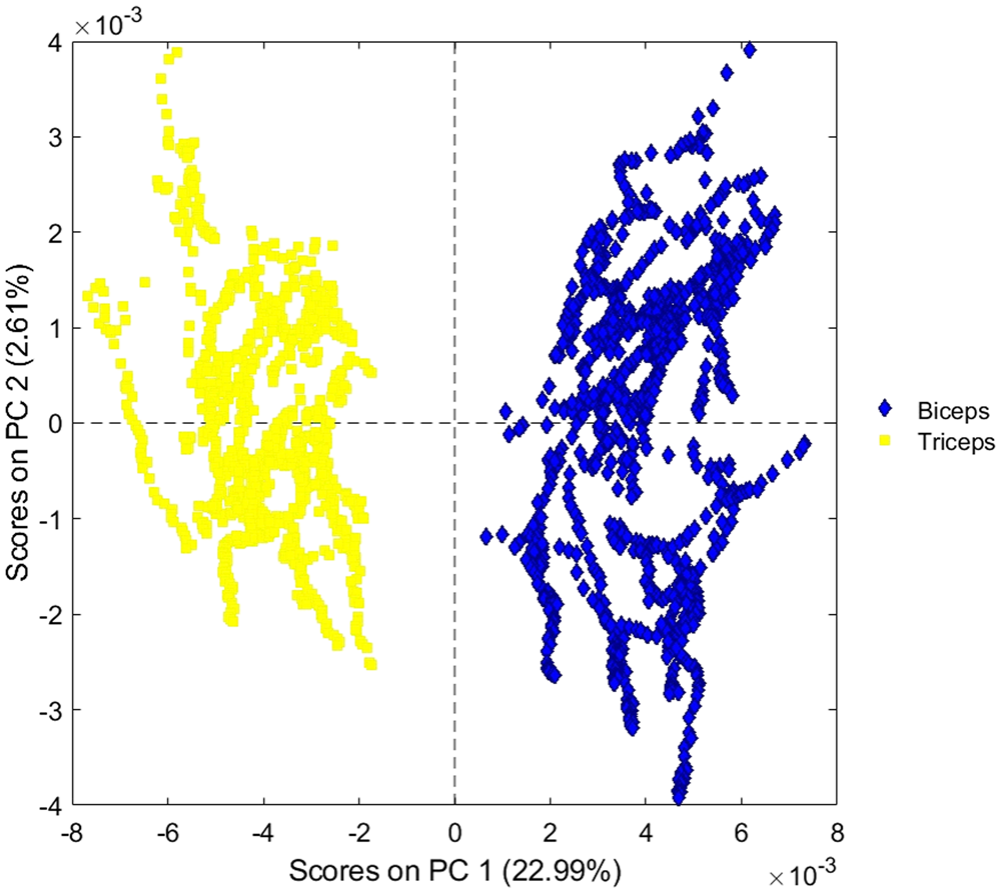
Scores plot of PC 1 vs. PC 2 of ventral arm/biceps (blue diamonds) and dorsal arm/triceps (yellow squares) reflectance data without any distinction among the different examined individuals.

Principal component analysis (PCA) was used to gather the spectra acquired from “ventral” and “dorsal” arms into two different clusters (Fig. 2). Principal component (PC) 1 resolved most of the variance between the two set groups.

### Correlation of anthropometric data with reflectance spectra

**Figure 3:**
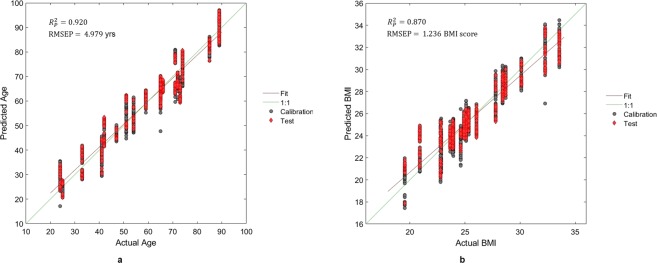
PLS regression results for the anthropometric variables, showing (**a**) Actual Age vs. Predicted Age and (**b**) Actual BMI vs. Predicted BMI. Each panel shows the squared correlation (${R}_{p}^{{\rm{2}}}$) and the Root Mean Square Error in Prediction (RMSEP) of each Y. The fit line is shown in red. The 1:1 line is shown in green. Grey dots represent the calibration data; red diamonds are the data used to validate the model.

The partial least squares (PLS) calibration using “age” values reached the coefficient of determination ${R}_{p}^{2}$ equal to 0.920 with a root mean square error in prediction (RMSEP) of 4.978 years (Fig. 3a). PLS using body mass index (BMI) values reached an ${R}_{p}^{2}$ of 0.870 and an RMSEP of 1.236 BMI units (Fig. 3b). Values for RMSEP, bias, R^2^ and RPD are shown in Table 1.

**Table 1:** Results of the PLS regressions.

Application	RMSEP*	Bias (Prediction)	R^2^ (Prediction)	RPD** (Prediction)
Age	5	0.010	0.920	3.54
BMI	1.24	−0.068	0.870	2.77

*RMSEP, Root Mean Square Error of Prediction; **RPD, Ratio of standard error of Performance to standard deviation.

**Figure 4:**
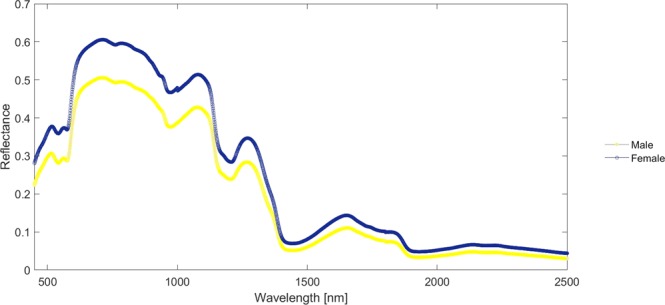
Mean reflectance spectra from ventral and dorsal arm for women and men.

**Figure 5:**
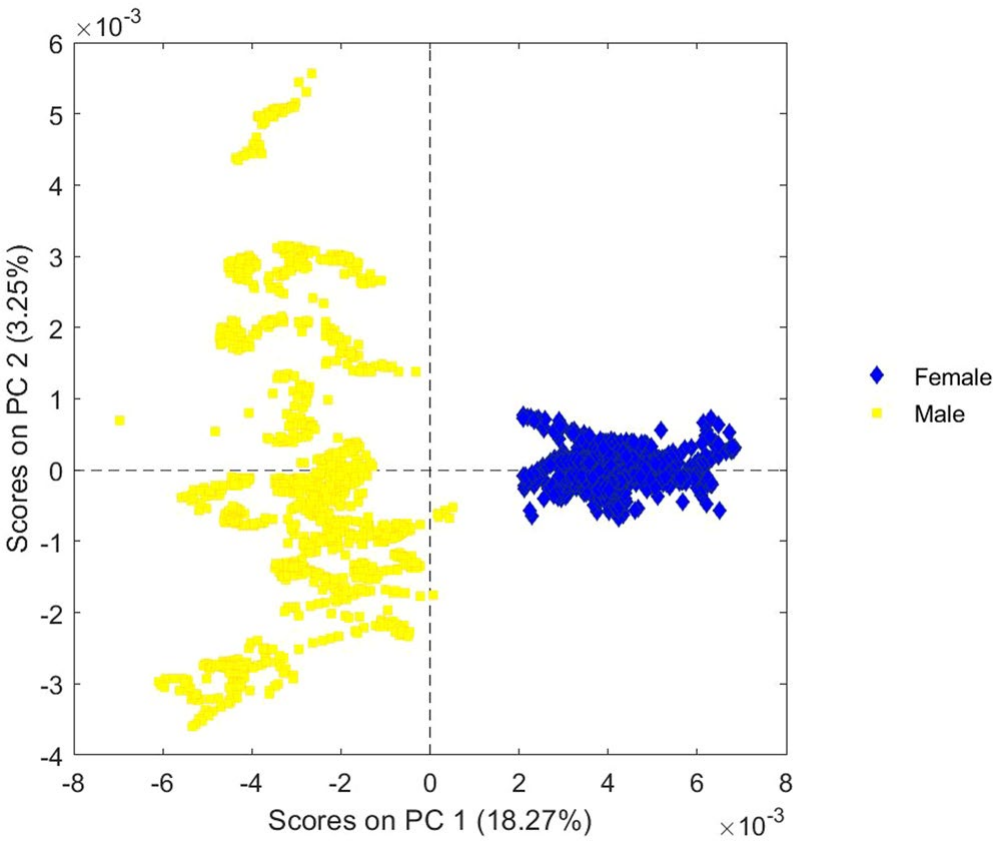
Scores plot of PC 1 vs. PC 2 of women (blue diamonds) and men (yellow squares) reflectance data.

**Figure 6:**
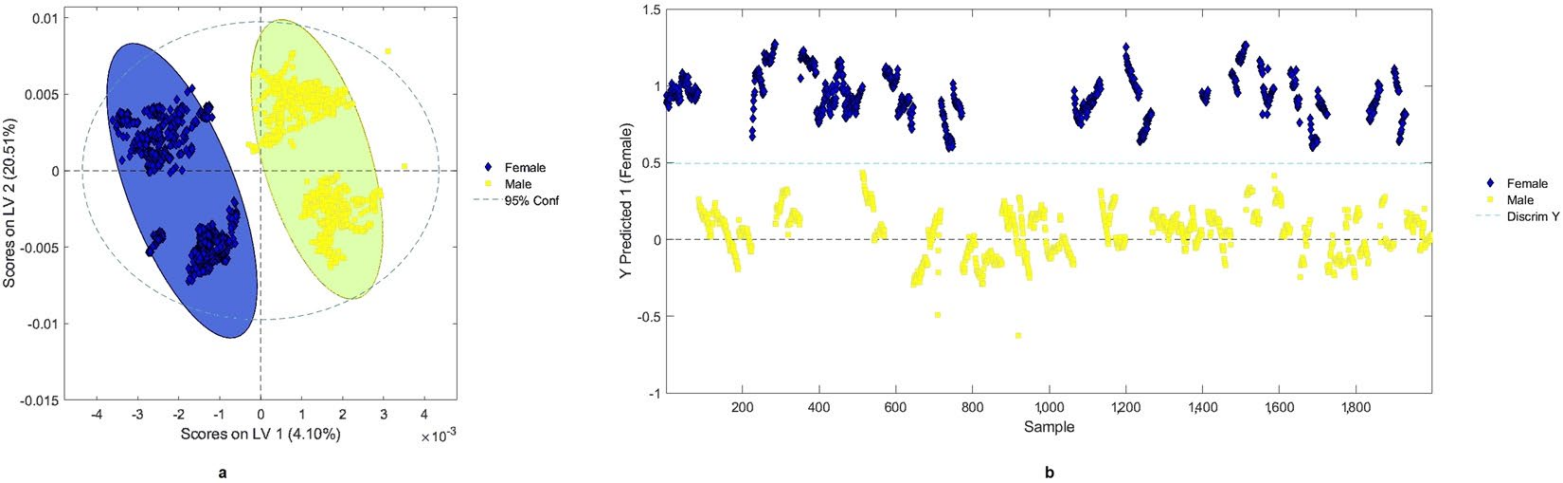
PLS-DA latent variables (LV) scores plot of LV 1 vs. LV 2 to discriminate sex from acquired spectra (**a**) and the position of the discrimination boundary for the two classes (“female” blue diamonds, and “male” yellow squares) as determined by PLS-DA model (**b**) for 450–2500 nm. Calibration and validation data are shown in both panels.

PCA was used to gather the spectra acquired from arms of “males” and “females” (Fig. 4) into two different clusters (Fig. 5). PC 1 resolved most of the variance between the two sexes. The partial least square discriminant analysis (PLS-DA) set up to discriminate subjects’ sex reached Sensitivity and Specificity proximal to 1, with ${R}_{p}^{2}$ of 0.898 for both male and female classes (Fig. 6).

### Classification models for discriminating dorsal and ventral aspects of the arm

**Figure 7:**
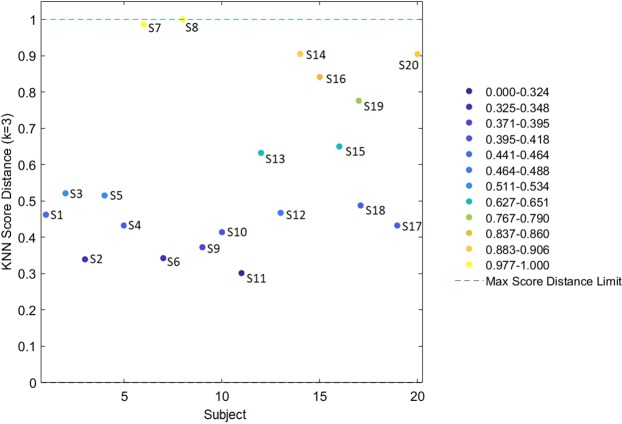
Subject *vs*. KNN Score Distance. Dots represent subjects according to the KNN score distance. Y values reflect the intra-subject variability of anthropometric variables.

The K-nearest neighborhood (KNN) plot score distance referred to the subjects was used to define the calibration set. Score plots of PLS-DA and the position of the discrimination boundary for the two classes “ventral arm” and “dorsal arm” were computed using a calibration set that employed a KNN score distance >0.433 (Fig. 7).

**Figure 8:**
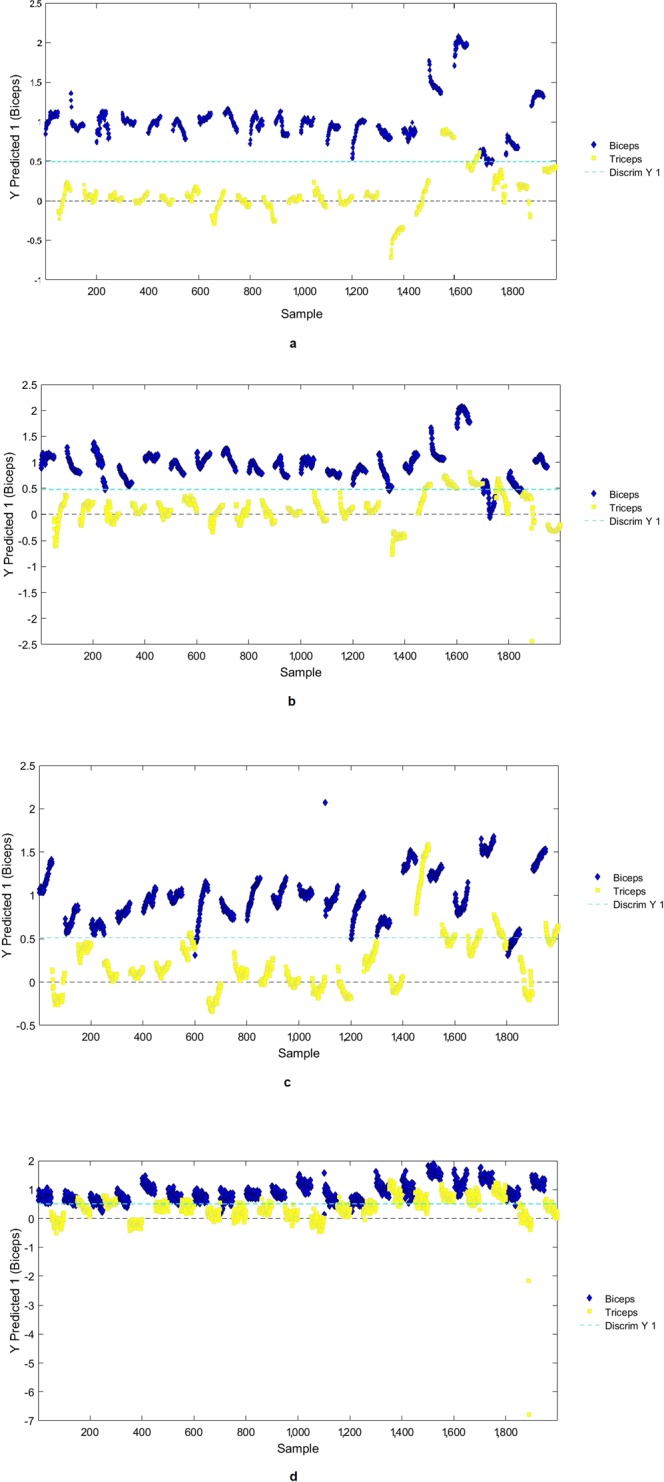
Position of the discrimination boundary for ventral arm/biceps (blue diamonds) and dorsal arm/triceps (yellow squares) as determined by PLS-DA model for: (**a**) 450–2500 nm; (**b**) VNIR/450–1000 nm; (**c**) SWIR1/1001–1800 nm; (**d**) SWIR2/1801–2500 nm. Scores lying above the boundary line are classified as “biceps”, Scores lying below the boundary line are classified as “triceps”. All panels display both the calibration and the test set.

Model’s classification performance was evaluated for each of the three spectral ranges analyzed by the three detectors embedded in the ASD FieldSpec®. Using the VNIR dataset, classification of the “ventral arm” had a sensitivity of 0.871 and a specificity of 0.595; using the SWIR 1 dataset, classification had a sensitivity 0.906 and a specificity 0.576; using the SWIR 2 dataset, classification had a sensitivity of 0.997 and a specificity of 0.338. Using the whole noise-cleaned spectral range of the instrument (450–2500 nm), classification had a sensitivity of 0.969 and a specificity of 0.799 (Table 2). The best overall accuracy was achieved by using the 450–2500 nm dataset, which reached an accuracy of 0.884, followed by SWIR 1 (1001–1800 nm) and VNIR datasets (400–1000 nm) which reached accuracies of 0.742 and 0.734, respectively. The SWIR 2 dataset (1801–2500 nm) resulted in the poorest classification performance, which reached an accuracy of 0.688. The positions of the discriminant boundaries (for Y predicted as “ventral arm”) as determined by the model for each dataset are shown in Fig. 8.

**Table 2:** Results of the PLS-DA classification models.

Detector’s spectral range	Class	Sensitivity (prediction)	Specificity (prediction)	Misclassification Error (prediction)	Precision (prediction)	Accuracy (prediction)
450–2500 nm (instrument whole spectral range)	Biceps	0.969	0.799	0.116	0.829	0.884
	Triceps	0.799	0.969	0.116	0.962	0.884
450–1000 nm (VNIR)	Biceps	0.871	0.595	0.266	0.684	0.734
	Triceps	0.595	0.871	0.266	0.821	0.734
1001–1800 nm (SWIR 1)	Biceps	0.906	0.576	0.258	0.683	0.742
	Triceps	0.576	0.906	0.258	0.858	0.742
1801–2500 nm (SWIR 2)	Biceps	0.997	0.338	0.332	0.602	0.668
	Triceps	0.338	0.997	0.332	0.992	0.668

## Discussion

This study investigated the Vis-SWIR reflectance spectra acquired in normal subjects *in vivo* from the skin over the ventral and dorsal aspects of the arm, aiming to highlight possible spectral signatures (i.e., *“fingerprints”*) related to the underlying muscle groups, the flexors (short and long heads of the biceps and brachialis from the ventral aspect) and the extensors (long, lateral, and medial heads of triceps from the dorsal aspect). NIRS proved a valuable tool to assess tissue optical properties *in vivo* non-invasively.

Biomedical optics provide the opportunity to study the interaction of light within materials, including biological tissues. In medicine, light has been used qualitatively by histologists to provide optical differentiation of cellular structures to aid in disease recognition, from 150 years ago. Development of small light sources, detectors, and fiberoptic probes made it possible to quantitatively measure these interactions, yielding diagnostic information at the structural, biochemical, and possibly pathophysiological level within intact tissues^28^.

Photons that penetrate tissues can be reflected or transmitted, and these two phenomena are attenuated mainly by absorption and scattering. Absorption may lead to a radiationless loss of energy to the medium, or induce either fluorescence or phosphorescence. Scattering refers to reflectance at unchanged frequency when it occurs in a stationary tissue, or when it is accompanied by a Doppler shift in the case of scattering by moving particles in the tissue (e.g., blood cells)^6^.

In the wavelength interval of 600–1000 nm, scattering predominates over absorption^28^. Because of low absorption, NIR light can propagate several centimeters, making it possible to extract information noninvasively from deep in organs^6^. Organ reflectance and scattering is the result of macroscopic differences in the refractive properties of tissues, and the microscopic heterogeneities of the refractive indices between extracellular, cellular, and subcellular tissue components. Changes in adsorption/reflection/scattering reflect biochemical and/or structural features, which in turn may be specific to anatomy, physiology, or pathology.

A huge amount of physical chemistry literature has focused on the re-emission of optical signals from tissues to describe changes in biochemistry detected by various spectroscopic approaches, and therefore to characterize quantitatively tissue constituents, components, structure, and pathology^2^. An accredited advantage of NIRS technology is the ability to build characteristic spectra that represent the “fingerprint” of the samples. Once subjected to statistical methods, these fingerprints offer the opportunity to understand the optical properties of the sample and classify them without the need for chemical information^9^. This was the precise aim of the present study, to acquire NIR reflectance spectra from living muscles, verify that they represent accurate and reproducible measures of the overall biological status of the muscle, that they change according to some anthropometric variables, and test they can be used to classify muscles. This is an NIRS application other than the real-time non-invasive detection of hemoglobin oxygenation in tissues.

In the present study, we collected reflectance spectra acquired from the ventral and dorsal surfaces of the arm. The arm is a heterogeneous structure that consists of layers with presumably different optical properties. It is covered by teguments (i.e., skin and appendages), has an adipose layer between the skin and muscles, and deeply houses a bone (*humerus*); all of these tissues are variably vascularized. NIR light has been shown to travel at least 10 cm through breast tissue, and 4 cm of skull/brain tissue or deep muscle using microwatt laser sources^29^; with higher power levels, light has been shown to penetrate through 7 cm of muscle and neonatal skull/brain^30^. As also shown by computed tomography studies confirming NIRS detection of hematomas located beneath the scalp surface^31^, the volume probed by NIR photons extends at least 4 cm beneath the arm surface; therefore, the collected spectra are supposed to represent average values over such a volume, including skin, fat, vessels, muscles, and possibly bone.

Skin is a highly heterogeneous tissue composed of multiple layers that differ in morphology and molecular composition. For NIRS applications it can profitably divided in three major layers of variable thickness. The inner layer is dermis (1 to 4 mm thickness), the outer layer is epidermis (thickness ranging from 40 μm in the eyelids to more than 1 mm on the palms), and the outermost layer is the *stratum corneum*, composed of flat terminally differentiated keratinocytes embedded in a matrix of lamellar lipids. *Stratum corneum*, is approximately 15 μm thick on most parts of the body, but it is thicker at the heel and palm, and thinner at the cheek^32^. The three skin layers have distinct and varying content of water; viable dermis and epidermis have almost constant content (around 80%), whereas *stratum corneum* has decreasing water content from the deeper part to the surface^33^. NIRS has long been used to measure skin layers water content^34^, and at present it is the only method to measure skin water mobility non-invasively^35^.

Intuitively, depth of NIR measurement is crucial when the target layer is the *stratum corneum* and the desired information is the water content - as it happens in dermato-cosmetic research - because inadvertent measurements below the outermost layer of the skin would overestimate water content due to the higher quantity found in epidermis and/or dermis. A series of elegant experiments^35–37^ showed that NIRS measurement depth in the skin varies depending on various factors. They include the geometry of the optical fiber probe used for radiating and detecting light (thick vs. thin probes), the clearance between the fiber-optic probe and the skin surface (to optimize skin specular reflection), and the wavelength of NIR radiation (because optical properties such as the absorption coefficient, the scattering coefficient, and the anisotropy parameter are functions of wavelength). By adopting specific arrangements of these three factors, it is possible to perform reliable NIR evaluation of the *stratum corneum*^35^. Reasonably, the system we used in the present study does not fulfill the optimal arrangement required for testing specifically the skin. First, the system is endowed with optical fiber probes having fixed geometry imposed by the manufacturer design (likely not optimized for skin measurements). Second, by eliminating clearance between the fiber-optic probe and the skin surface, on the one hand we prevented skin specular reflection, on the other we promoted longer measurement depths. Third, the light radiation emitted by the probe ranged from 450–2500 nm wavelength, a wider range than that usually analyzed in dermatologic NIRS. In addition, while NIR measurements of the *stratum corneum* thickness are available for the forehead, cheek, jaw, elbow, volar forearm, palm, knee, and heel^37^, to our knowledge no specific data exist that differentiate the skin areas contacted by the probe in our investigation (ventral and dorsal arm). Finally, no humectant substance was present on the skin other than subjects’ natural moisturizing factor. Therefore, we consider unlikely that the differences we found in the spectra collected from dorsal and ventral arm may have stemmed from differences in the correspondent skin probed by NIR radiation.

Because adipose tissue has no precise oriented structure, and in a healthy subject the structure of vessels and bone is reasonably invariable when probed from the ventral or dorsal aspect of the arm, we reasoned that the differences observed between the respective spectra could be ascribed to the underlying muscles, the only structures that exhibit a high degree of spatial organization. Skeletal muscle is indeed made up of a grouping of muscle fibers surrounded and supported by connective tissues; muscle fibers are tridimensionally uniform, being long (1–40 mm), cylindrical (10–100 mean diameter) and folded in bundles that may extend parallel or with a certain angle with the muscle itself. At a microscopic level, the orientation of fibers is known to affect the optical properties of muscle^38,39^. The substances primarily responsible for absorption of light (known as endogenous chromophores) are oxy- and deoxyhemoglobin, melanin, myoglobin, and water. These likely contribute an essentially constant background absorbance in the same organ lighted either from the top or the bottom^40^. In addition, previous investigators studying *in vivo* time-resolved reflectance spectroscopy of muscles acquired spectra from the skin surface overlying the examined muscle^41,42^. Interestingly, in the study that aimed to evaluate *in vivo* the optical properties of different biological tissues (arm muscle, abdomen, and forehead), the absorption spectra of the “arm muscle” were acquired from the “bicipital region”. Finally, the wavelengths of reflectance minima that characterize our data are located substantially in the same position as the absorbance peaks reported in studies aiming to classify meats from various cuts or animals^9,14^. Reasonably, our spectra mainly reflect the optical properties of muscles: ventral arm spectra originated primarily from the “biceps”, and dorsal arm spectra primarily from the “triceps”.

The shape of the reflectance spectra grand averages of biceps and triceps (i.e., their fingerprints) shows marked differences around 760 nm, 970 nm, 1200 nm, and at 1440 nm (Fig. 1b). These wavelengths were associated with the reflectance minima, which distinguish biceps from triceps corresponding to specific adsorbing groups: H_2_O, CH, CH_2_ (762 nm); H_2_O, CH (973); CH (1206 nm); H_2_O, CH, ROH, CONH_2_, CONHR (1442 nm); CH (1796 nm); H_2_O, RCO_2_R, CONH_2_ (1930 nm); and RNH_2_, CHC, CC (2186 nm). Absorption of molecules in the NIR region results from the absorption of overtones and combination of stretching-bending vibrations of atomic groups such as CH, OH, and NH, which contain hydrogen atoms. Overtones and combination bands are types of vibrations related to the fundamental vibrations seen in the infrared region. Overtones occur at about two or three times the frequency of the fundamental vibration, and increasing overtones cause absorption intensity to decrease. In contrast, combination bands are the sum of several fundamentals from different vibrations, which are typically found at lower energies than overtones^2^. The peak wavelengths we found are similar to those reported in studies about the classification of various meat cuts or meats from various animals^9,14^; 762 nm is the wavelength at which the absorbance peak is “more pronounced in muscles than in the other tissues” (e.g., the forehead and abdomen)^42^, and is considered produced by the oxidation of myoglobin (deoxymyoglobin)^4^. On this basis, we consider it likely that differences in NIRS fingerprints that distinguish biceps from triceps reasonably reflect intrinsic biological features that are specific to muscle tissue.

A first hypothesis that may explain the specificity of biceps and triceps fingerprints shown by chemometric analysis and predictions of the model is the macroscopic arrangement of muscle fibers, known as muscle’s architecture. Skeletal muscle architecture is defined as “the arrangement of muscle fibers within a muscle relative to the axis of force generation”^43^. Although many architectural arrangements can be found in the animal kingdom, a plausible simplification allows to consider three general classes of muscle fiber architecture^44^. The first is the parallel or longitudinal architecture, which pertains to muscles composed of fibers that extend parallel to the muscle’s force-generating axis; the second is the unipennate architecture, typical of muscles with fibers that are oriented at one angle relative to the force-generating axis. The third and most general class is that of multipennate muscles, composed of fibers oriented at several angles relative to the axis of force generation. Because the biceps has a parallel architecture^44^, whereas the triceps has a bipennate architecture^45^, their fiber arrangement may be one factor determining the NIRS fingerprint.

Aside from pennation angles, other important parameters of muscle architecture are muscle fiber length (physiologically related to the maximum muscle excursion), and muscle physiologic or anatomic cross-sectional area (a ratio of the muscle volume divided by the fiber length, expressed as units of area), physiologically related to the maximum muscle force^44^. Apart from the methodological considerations pertaining to the evaluation of these parameters, cadaver studies found that biceps has greater fiber lengths than any portion of the triceps^46^, and *in vivo* MRI studies showed that cross-sectional area differs between biceps and triceps^47^. To conclude, the architecture of a given muscle is extremely consistent between individuals of the same species, giving rise to the concept that certain constraints are present that determine the architectural properties of muscle^44^. Muscles with large physiological cross-sectional areas have a large number of sarcomeres lying in parallel; in contrast, long-fibered muscles have more sarcomeres in series^48^. In addition to conditioning muscle’s contractile properties, the peculiar spatial arrangements that determine the muscle architecture also reasonably affect the optical properties of muscle, and therefore may be summarized and surrogated by NIRS fingerprints. This idea finds support in animal studies using diffusion tensor imaging at 4.7 T; these studies detected significant differences in anisotropy, spatial variations of anisotropy, and fiber tract orientation between muscles^49^.

Most muscles contain a mixture of muscle fiber types which can be distinguished by biochemical and histochemical methods that allow visualization of enzyme content, metabolic substrates, or structural proteins within individual fibers^50^. The differences in the biochemical profile of the constituent muscle fibers are the basis of the physiological differences between motor units and muscles. On the basis of the content of metabolic enzymes (which suggests preferential use of particular metabolic pathways), capillary supply, structural proteins, and both nerve and muscle fiber diameter, predominantly glycolytic (fast twitch) and predominantly oxidative (slow twitch) muscles are distinguished. The relative percentage of slow and fast fibers, known as fiber composition, differs in individual muscles and is subject to various modulating factors such as the type of motor exercise, immobilization, or disease. Although there are known variations between individuals, classical autopsy studies showed definitively that both surface and deep components of the biceps are composed of relatively fewer fast-twitch predominant muscle fibers than corresponding components of the triceps^51,52^.

Fiber composition affects important structural aspects of muscles. Whereas there is usually one capillary at each corner of the cell in predominantly glycolytic (fast twitch) muscle, in predominantly oxidative (slow twitch) muscles additional capillaries are located at the midpoint along the endomysial septum that joins the corners of cells^50^. This observation points toward a further possible contributor to muscle fingerprints: the intramuscular connective tissue. This tissue provides the pattern of the muscle’s internal organization into fascicles and fibers, and is easily visualized upon dissection. Individual muscles are enveloped by the epimysium, fascicles within the muscle by the perimysium, and individual muscle fibers by the endomysium. These three intramuscular connective tissue structures generally differ in composition and structure both within and between muscles, and they have a number of functions. They provide mechanical support for the major nerves and blood vessels servicing the tissue as a whole, as well as for individual neurons and capillaries for each muscle fiber; they signal cell–matrix interactions during muscle proliferation and growth; and they have both passive elastic and active dynamic actions, which contribute to the mechanical properties of muscle. Interestingly, the timing and rates of expression of the connective tissue proteins that compose intramuscular connective tissue differ between muscles^53^.

PCA shows that muscle fingerprints are well clustered (Fig. 2), with the scores of spectra from biceps clearly separated from those from triceps. PC 1 resolved most of the variance between the two set groups.

**Figure 9:**
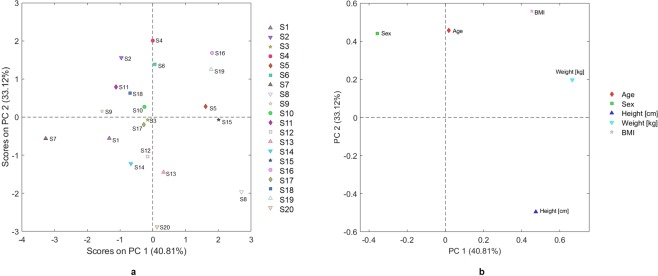
(**a**) Scores plot and (**b**) loadings plot of PC 1 *vs*. PC 2 values calculated on anthropometric variables. The positions of the anthropometric variables in the loadings plot indicate which variable weights the most in the score of each subject (shown in scores plot).

Anthropometric variables may affect reflectance spectra, and scores and loadings plots related to the discrete data (Fig. 9a,b, respectively) support our hypothesis. Reasonably, anthropometric variables relate to the general body constitution, which in turn determines cutaneous and subcutaneous layer thickness and relative composition in terms of fibrous, muscular, and adipose tissue. Because the fibrous and adipose tissue content of a muscle depends on the amount (and/or composition) of the overlying subcutaneous layer^54^, which in turn is related to total body adiposity, we conclude that muscle fingerprints reflect factors that act to determine each subject’s anthropometric variables. This conclusion is supported by the results of PLS regression using reflectance spectra to predict anthropometric variables such as “age” and “BMI” (Fig. 3a,b), as well as the results of PLS-DA procedure to discriminate the sex of the subjects (Fig. 6). Therefore, we plan further studies aimed to acquire extensive descriptors of anthropometrics (including plicometry, layer thickness measurements, and measures of the individual body segment studied) to unravel the precise relationship between muscle NIRS fingerprints and anthropometric variables.

In the present study, we used preprocessing methods that do not require a priori information to calibrate for inter-patient and intra-patient variation in tissue reflectance spectra; we applied PCA to dimensionally reduce each type of preprocessed spectral data with minimal information loss; and finally, we developed a probability-based classification algorithm using logistic discrimination (PLS-DA), a supervised technique for pattern recognition^55,56^. The best classification results (with reference to the 3 investigated spectral ranges as collected by the 3 detectors inside the ASD FieldSpec®) occurred using VNIR and SWIR1 range datasets, which achieved the highest sensitivity and specificity and the best precision and accuracy values (Table 2). The predicted identity of muscle from the multivariate model achieved an equivalent accuracy to the conventional clinical examination. These very good classification results demonstrate that the procedure is reliable and that classification models are robust. Similar discrimination results using PLS and the raw spectra were reported previously by other authors studying the authenticity of meats^9,57^.

Similar to the spectroscopic techniques applied to authenticity issues, our spectral data are nonselective. Our reflectance spectra do not contain information about the intimate composition of the material under analysis, rather they yield structural information that constitutes the fingerprint of the sample^58,59^. Because the philosophy of discriminant techniques is based on the fundamental assumption that the spectra of samples of a given material are similar and different from spectra of other materials when the raw spectra are compared^58^, the information used for identification purposes by our model came from the overall structural muscle composition that constitutes the fingerprint of the sample (i.e., intra-muscular fat and connective tissue, capillary supply, type of muscle fibers, and resulting muscle structure and architecture). The notions that the ability of an NIR-based model to discriminate species derives from the vibrational responses of chemical bonds to near-infrared radiation and that the accuracy of the model increases with variability in these chemical entities responding to the near-infrared range of the electromagnetic spectrum^60^ lead us to conclude that the sample fingerprints we acquired from the upper limb are robust and reliable markers of the biological and physical state of the underlying muscles.

Overall, the present findings describe the feasibility of using VNIR optical spectroscopy to analyze human tissues *in vivo*. The exploratory study conducted here showed that NIRS identifies muscles’ optical fingerprints from body segments, and that a model can be designed to individuate muscles starting from those fingerprints. Practically, VNIR spectroscopy distinguishes flexors from extensors in the upper limb, thereby proving effective as an objective indicator of the current state of a muscle that can be measured accurately and observed reproducibly. Because of the lack of non-invasive *in vivo* analysis of human muscles, and due to the favorable properties of portable spectrometers (i.e., size, speed of analysis, and pain-free approach), VNIR and NIR analysis may represent a new clinical/research tool that can be added to standard clinical investigation without significant cost and time penalties. An objective and reproducible surrogate marker of the current biological state of a muscle that is easily obtainable at bed-side may enable more thorough evaluation of the muscular system in normal subjects and patients, may ultimately contribute to distinguish healthy status from disease, may help with non-invasive follow-up in muscle diseases, and may optimize the monitoring of therapeutic or rehabilitative interventions’ effectiveness. We have planned additional studies to explore the reliability of NIR fingerprints in greater detail when varying muscle physiologic variables or testing pathological conditions.

## Materials and Methods

### Subjects

Study participants were enrolled at the Department of Medical-Surgical Sciences and Biotechnologies, among relatives and care-givers of patients coming to the Academic Neurology Unit. Participants were selected according to the following criteria: no history of skin or musculoskeletal abnormalities involving the upper limb, no abnormal color of the skin (including tan), no sign of skin disease (including hypo- or hyperhidrosis), no sign of neurological condition other than sporadic episodic headache, no systemic condition, and not taking any drug known to induce secondary muscle abnormality. Hypertension and dyslipidemia were not considered exclusion criteria. None of the participants made use of moisturizing creams over the skin of the arms.

Following these criteria, 20 Caucasian, southern European healthy subjects (age 25–89 years, 10 women) were recruited. All study participants provided written consent before being included in the study, which was approved by the institutional review board (Comitato Etico Lazio 2, protocol number 0167183/2018). All methods were carried out in accordance with the relevant guidelines and regulations.

Demographic (sex, age), clinical (drugs and/or conditions), and anthropometric data (height, weight, BMI) were collected for each subject (Table 3).

**Table 3:** Demographic, clinical and anthropometric data of study participants.

Subject	Age [years]	Sex	Weight [Kg]	Height [cm]	BMI [kg/m^2^]	Hypertension*	Dyslipidemia*	Headache*
S1	41	F	66	170	22.84	0	0	0
S2	76	F	70	162	26.67	1	1	0
S3	89	M	70	171	23.94	1	1	0
S4	71	F	81	163	30.49	1	1	0
S5	73	M	81	175	26.45	1	1	0
S6	65	F	80	167	28.69	0	0	0
S7	47	F	50	160	19.35	0	0	1
S8	33	M	95	191	26.04	0	1	0
S9	51	F	65	165	23.88	0	1	1
S10	85	M	73	167	26.18	1	1	0
S11	65	F	67	165	26.61	1	1	0
S12	54	M	71	172	24	1	1	0
S13	59	M	73	179	22.78	0	1	0
S14	24	M	67	165	24.61	0	1	0
S15	74	M	91	180	28.09	1	1	0
S16	42	F	98	170	33.91	0	0	1
S17	66	M	70	167	25.1	1	1	0
S18	72	F	72	170	24.91	0	1	0
S19	65	M	91	167	32.63	1	1	1
S20	25	M	73	183	21.8	0	0	0

*Affected: No = 0, Yes = 1.

### Portable spectroradiometer system

The ASD FieldSpec® 4 Standard-Res (ASD Inc., Boulder, CO, U.S.A.)^61^ is a portable spectroradiometer that works in the spectral range of 350–2500 nm and has a spectral resolution of 3 nm at 700 nm and 10 nm at 1400/2100 nm^62^. This instrument essentially consists of a detector case and a fiberoptic cable with a contact probe, connected to a laptop computer. Inside the detector case, the system has different separate holographic diffraction gratings with three separate detectors. Order separation filters cover each detector to eliminate second and higher order light. The detector system is made up of a VNIR detector (350–1000 nm; 512 element silicon array), the SWIR1 detector (1001–1800 nm; Graded Index InGaAs. Photodiode, Two Stage TE Cooled), and the SWIR2 detector (1801–2500 nm; Graded Index InGaAs. Photodiode, Two Stage TE Cooled)^61^. The ASD Contact Probe consists of a halogen bulb light source with a color temperature equal to 2901 ± 10%°K. It has a length of 25.4 cm (including the probe grip) and it has a weight of 0.7 kg. The light source is placed at 12° from the normal axis to the contact probe spot plane (light source angle). The fiber optic head is placed at 35° from the normal axis to the contact probe spot plane (measurement angle). Since the spot size of the contact probe is 10 mm, the Field of View (FOV) is about 1 cm^2^ ^63^. The native software of the ASD instrument, called RS3, has been used for data acquisition.

### Instrument calibration and hyperspectral data handling

The spectroradiometer calibration procedure started with dark acquisition (*D*_*i*_) and the measurement of “white reference” (*W*_*i*_) material^64^. After this calibration stage, the spectra (*R*_0*i*_) were acquired and the reflectance (*R*_*i*_) computed using the Eq. 1:

1 $${R}_{i}=\frac{{R}_{0i}-{D}_{i}}{{W}_{i}-{D}_{i}}$$

Calibration was conducted using ASD RS3 software. The dark reference was generated by the software as a current calibration file for the dark reference. The white reference was acquired on a Spectralon white reference standard from LabSphere™. Instrument “.asd” data files were converted into ASCII text files with the aid of ViewSpec Pro Ver. 6.2.0. software. ASCII (“.txt”) text files were imported into the MatLab® environment (MATLAB R2016b; ver. 9.1.0.441655), using “fieldspec_import.m”, an ad hoc script written for this purpose. Imported data files were analyzed using the Eigenvector Research, Inc PLS_toolbox (ver. 8.2.1) operating inside the MatLab® environment. Data were stored in datasets; objects and classes were set.

### *In Vivo* spectra acquisition

Vis-IR reflectance spectra acquired from the ventral and dorsal aspects of the arm were collected using the ASD FieldSpec® 4 Standard-Res field portable spectroradiometer. A health professional (ACa) cleaned both the probe and the skin contact zone with disposable skin cleaning wipes (Amukine). The same health professional adjusted the length of the holder’s extensible rod in order to place the instrument contact probe on the subject’s limb skin, and an engineer (RG) controlled the spectroradiometer from a remote laptop. The position of the contact probe for spectra collection was standardized according to positions of the motor points^65^. The muscle motor point, also known as the motor entry point, represents the location where the motor branch of a nerve enters the muscle belly. Spectra were acquired with the muscle at rest, the segment fully supported, and the limb held in fixed posture (elbow angle at 90°). To increase the precision and accuracy of data collection, 50 spectra were acquired from each contact point (50/side/subject, for 100 spectra/subject, and 2000 spectra in the study). The duration to acquire single spectra was approximately 2 seconds; therefore, the time needed to acquire a total of 50 spectra/side/subject was approximately 100 seconds.

### Spectra preprocessing

To remove the noisiest part of the spectra, before starting the chemometrics analysis, the main dataset (n = 2000) was reduced from 350–2500 nm to 450–2500 nm.

To remove physical phenomena in the spectra in order to improve the subsequent multivariate analysis (including scatter-correction and spectral derivatives), a selected combination of preprocessing steps was performed using Extended Multiplicative Scatter Correction (EMSC), Generalized Least Square - Weighting (GLS-W) on classes (with α = 0.002, to increase the filtering effect) and Mean Center (MC) algorithms. EMSC was used to correct scatter artifacts^66^. GLS-W, which consists of a multivariate filter that calculates a filter matrix based on differences among groups of otherwise similar samples^67,68^, was applied to down-weight the difference or “clutter” inside classes. Finally, the MC was applied to remove the mean value from the data and to enhance differences among samples.

### Principal component analysis

The exploratory analysis of reflectance spectra data was performed using PCA, a mathematical procedure designed to resolve sets of data into orthogonal components whose linear combinations approximate the original data to any desired degree of accuracy^67,68^. PCA makes it possible to extract the dominant patterns from the reflectance spectral dataset matrix, in terms of the product of two smaller matrices of scores and loadings^69,70^. Two PCs were chosen by exploring the eigenvalues plot; outliers were identified and excluded by exploring Hotelling’s T2 vs. Q Residuals plots.

A separate PCA was performed on subjects’ anthropometric data. We built a dataset that included information about age (value), sex (added in binary logic, using: “0 = male” and “1 = female”), weight (kg), height (cm), and body mass index (BMI). Data were preprocessed using the Autoscale algorithm, one of the most widely used preprocessing techniques^71^. Five PCs were used to perform PCA on the discrete data.

### Partial least squares regression

The PLS regression combines features of both multiple regression and PCA^72^. The main objective of PLS regression is to predict the matrix *Y* that stores the dependent variables from *X*, the matrix containing the predictors, and to describe the underlying common structure^73^. PLS constructs a linear regression model by projecting the predictor variables and response variables to a new set of latent variables (LVs), the covariance of which is maximized^55^. This technique finds broad application in various fields of neuroscience^74^ and chemometrics^75^.

PLS modelling was used to evaluate correlation between spectra (n = 2000) and anthropometric variables such as BMI and age. Using the Kennard-Stone algorithm, 70% of the spectral samples were randomly selected to build up a calibration set; the remaining 30% were used for validation. Two models were computed, one using age as *Y* and the other using BMI values from each subject. Cross-validation was performed using venetian blinds with 5 splits and 1 sample per split in each model to tune the calibrations^76,77^. Two LVs were chosen for the PLS of “Age” values, and 3 LVs were chosen for the PLS of “BMI” values.

Goodness of fit of the spectral data in the regression model was assessed with two main parameters: RMSEP, and ${R}_{p}^{2}$ ^72^. In order to compare and evaluate the predictive ability of the models values for *R*^2^, bias, and RPD were assessed.

### Partial least square discriminant analysis

To classify and predict the two classes of muscle groups, we used PLS-DA, a statistical method that explores the predictive models between predictor and response variables^55,73^. It is essentially an inverse-least square approach to the linear discriminant analysis, another multivariate inverse least squares discrimination method. In PLS-DA, the PLS regression is utilized to develop a model able to predict the class number for each sample under study^77,78^. To evaluate the performance of the classification model, we used the confusion matrix with commonly used performance metrics: Precision, Accuracy, Misclassification Error, Sensitivity, and Specificity^79,80^.

To discriminate individual spectra acquired from the dorsal and ventral arm, the calibration set was built by adopting the KNN score distance, rooted to the PCA performed on the anthropometric data of the individual objects of the investigation^81^. The KNN score distance is essentially a measure of the average distance to the *k* nearest neighbors in score space for each sample. Using this procedure, a more reliable calibration set is obtained, that chooses the tails of the sample distribution [i.e., samples with KNN score distance (*k* = 3) > 0.433]. Hence, spectra collected from 14 subjects (70% of total spectra, n = 1400) were selected by the algorithm and included in the calibration set, whereas spectra from the remaining 6 subjects (30% of total spectra, n = 600) were included in the validation set. This PLS-DA model was subjected to the Venetian Blinds cross-validation method, and two LVs were chosen using the whole noise-cleaned instrument spectral range (450–2500 nm).

Separate PLS-DA classification models were set up based on single-detector spectral range subsets. According to the detecting features of the 3 sensing units embedded inside the ASD FieldSpec® device, the raw dataset (450–2500 nm) was split into three parts: VNIR (450–1000 nm), SWIR 1 (1001–1800 nm), and SWIR 2 (1801–2500 nm). Each split dataset underwent the same procedure described beforehand (i.e., 70% of the preprocessed dataset of individual spectra for each spectral region - VNIR, SWIR 1 and SWIR 2 - included in the calibration set; the remaining 30% of total spectra included into the validation set). These PLS-DA models were subjected to the Venetian Blinds cross-validation method; 2, 3 and 8 LVs were selected to model VNIR, SWIR 1, and SWIR 2 datasets, respectively.

A PLS-DA was applied to the preprocessed dataset of individual spectra (n = 2000, 450–2500 nm spectral range) in order to discriminate the subjects’ sex. The calibration set was built by randomly selecting 70% of the preprocessed spectral samples with the Kennard-Stone algorithm. To assess the optimal complexity of the model, and to select the number of LVs, we used Venetian Blinds as a cross-validation method^76^. Two LVs were chosen, and the model was validated using the remaining 30% of the preprocessed dataset of individual spectra.

## Data Availability

The datasets generated during and/or analysed during the current study are available from the corresponding author on reasonable request.
